# Incomplete Radiofrequency Ablation Enhances Invasiveness and Metastasis of Residual Cancer of Hepatocellular Carcinoma Cell HCCLM3 via Activating β-Catenin Signaling

**DOI:** 10.1371/journal.pone.0115949

**Published:** 2014-12-26

**Authors:** Ning Zhang, Lu Wang, Zong-Tao Chai, Zi-Man Zhu, Xiao-Dong Zhu, De-Ning Ma, Qiang-Bo Zhang, Yi-Ming Zhao, Miao Wang, Jian-Yang Ao, Zheng-Gang Ren, Dong-Mei Gao, Hui-Chuan Sun, Zhao-You Tang

**Affiliations:** 1 Liver Cancer Institute and Zhongshan Hospital, Fudan University, Key Laboratory for Carcinogenesis and Cancer Invasion, The Chinese Ministry of Education, Shanghai, P. R. China; 2 Department of Hepatobiliary Surgery, The First Affiliated Hospital of Chinese PLA General Hospital, Beijing, P. R. China; 3 Department of General Surgery, Qilu Hospital, Shandong University, Jinan, P. R. China; University of Hong Kong, Hong Kong

## Abstract

**Background:**

Radiofrequency ablation (RFA) is one of the curative therapies for hepatocellular carcinoma (HCC), however, accelerated progression of residual HCC after incomplete RFA has been reported more frequently. The underlying molecular mechanism of this phenomenon remains to be elucidated. In this study, we used an incomplete RFA orthotopic HCC nude mouse model to study the invasive and metastatic potential of residual cancer as well as the correlated mechanism.

**Methods:**

The incomplete RFA orthotopic nude mouse models were established using high metastatic potential HCC cell line HCCLM3 and low metastatic potential HCC cell line HepG2, respectively. The changes in cellular morphology, motility, metastasis and epithelial–mesenchymal transition (EMT), and HCC cell molecular markers after *in vitro* and *in vivo* incomplete RFA intervention were observed.

**Results:**

Pulmonary and intraperitoneal metastasis were observed in an *in vivo* study. The underlying pro-invasive mechanism of incomplete RFA appeared to be associated with promoting EMT, including down-regulation of E-cadherin and up-regulation of N-cadherin and vimentin. These results were in accordance with the *in vitro* response of HCC cells to heat intervention. Further studies demonstrated that β-catenin was a pivotal factor during this course and blocking β-catenin reduced metastasis and EMT phenotype changes in heat-treated HCCLM3 cells *in vitro*.

**Conclusion:**

Incomplete RFA enhanced the invasive and metastatic potential of residual cancer, accompanying with EMT-like phenotype changes by activating β-catenin signaling in HCCLM3 cells.

## Introduction

Hepatocellular carcinoma (HCC) is the fifth most common cancer worldwide and the third leading cause of cancer-related death [1]. Although surgical resection is the standard treatment modality for HCC, its use is usually limited because the majority of patients, even with small HCC, have associated severe liver dysfunction [2], [3]. Liver transplantation provides an alternative treatment for small unresectable HCC, however, the shortage of liver grafts limits the applicability of this approach [4]. Due to these circumstances, several non-surgical techniques have been introduced for HCC treatment, such as radiofrequency ablation (RFA), percutaneous ethanol injection (PEI) and microwave coagulation therapy (MCT) [5], [6]. Among these techniques, RFA is currently the most widely used treatment option due to its simplicity, safety, minimal invasiveness, repeatability and shorter hospital stays [4]. RFA is considered the best option for unresectable HCC in patients with no more than three liver nodules, a maximum 3 cm diameter tumor, and with preserved liver function (Child-Pugh A and B) [7], [8]. However, one of the major challenges with RFA is residual tumor tissue and local recurrence after local treatment, it was reported that the post-RFA recurrent rates range from 49 to 74% [9]–[11]. Moreover, the local recurrent tumor after RFA showed a more invasive growth, more vascular invasion and less differentiation compared with tumors of patients without RFA [12].

The Wnt/β-catenin pathway is an important signaling pathway in HCC [13], [14]. It has been reported that one third of HCC are associated with aberrant expression of β-catenin [15]. β-catenin (CTNNB1), is a central molecule in the Wnt signaling pathway, and is a multifunctional protein involved in cell-cell adhesion, signal transduction, cellular differentiation regulation and proliferation. Our team had proven that hypoxia could induce β-catenin overexpression and/or intracellular accumulation in four HCC cell lines through down-regulating the endogenous degradation machinery, and hypoxia can also enhanced invasiveness *in vitro* and metastasis *in vivo* for Hep3B and MHCC97 cells [16]. Mutations in the Wnt/β-catenin pathway members also result in aberrant activation of the target genes, including those encoding for activators of epithelial–mesenchymal transition (EMT) [17]. EMT plays a pivotal role in the critical phases of embryonic development and contributes to physiological processes, such as tissue repair, regeneration and pathological conditions, including carcinogenesis and fibrosis. In addition, it also participates in intrahepatic dissemination and distant metastasis during HCC progression [18]–[21]. β-catenin plays a crucial role in the onset and progression of EMT. Several studies have demonstrated the relationship between β-catenin signaling and EMT [22]. Oh et al. reported that β-catenin activation caused induction of Snail and ZEB1 expression, mesenchymal cell marker expression and repression of E-cadherin expression in some epithelial cells during the pathogenesis of adenomyosis [23]. Zhao et al. provided evidences that Wnt/β-catenin signaling pathway directly involved in EMT induced by HIF-1α, and blocking Wnt/β-catenin signaling pathway caused reversal of EMT and metastatic phenotypes changes [24]. However, the role of β-catenin in residual cancer after incomplete RFA is still unclear. In this study, we observed that incomplete RFA enhanced the invasive and metastatic potential of residual cancer, and examined alterations in β-catenin expression in incomplete RFA tumor tissues *in vivo* and in heat-treated HCC cells *in vitro*. Furthermore, we evaluated its significance in metastatic phenotype changes induced by heat intervention.

## Materials and Methods

### Cell Culture and Animals

HCCLM3 cells with high metastatic potential and HCCLM3-G cells which were HCCLM3 cells transfected with green fluorescence protein, HepG2 cells with low metastatic potential and HepG2-G cells which were HepG2 cells transfected with green fluorescence protein, were used in *in vitro* and *in vivo* experiments, respectively (established at the Liver Cancer Institute, Zhongshan Hospital, Fudan University, Shanghai, China) [25]. The cells were maintained in Dulbecco's modified Eagle's medium (DMEM, Gibco BRL, Rockville, MD, USA) with 10% fetal bovine serum (Life Technologies, Carlsbad, CA, USA), 100 U/mL penicillin at 37°C in a humidified atmosphere containing 5% CO_2_. Male BALB/c nu/nu mice, weighing 18–20 g at 4–6 weeks of age, were obtained from SLAC Laboratory Animal Co, Ltd, Shanghai, China. All mice were maintained under specific pathogen free conditions. All animal protocols were approved by the Ethical Committee on Animal Experiments of Animal Care Committee of Fudan University. All efforts were made to minimize suffering of experimental animal (S1 Checklist).

### Equipment and Establishment of Incomplete RFA Orthotopic Nude Mouse Model

The main equipment used were comprised of the RITA medical system model 1500X RF generator (RITA Medical Systems, Fremont, CA, USA), retractable multiple hook RFA needle (S1A Fig.) (RITA Star Burst XL, Angio-Dynamics, Latham, NY, USA), and RITA grounding pads (Angio-Dynamics).

The human HCC orthotopic nude mouse models with HCCLM3-G cells and HepG2-G cells were established as previously described [26]. The nude mice were randomly divided into the incomplete RFA group (n = 12) and the control group (n = 12). Two weeks after orthotopic implantation, the incomplete RFA was performed as follows: after anesthesia by Pelltobarbitalum Natricum with administered by intraperitoneal injection (50 mg/kg) , the animal was placed on a conductive metal plate with the limbs fixed and RITA grounding pads were adhered to the back of this metal plate to ensure good electrical conductivity. Next, the abdomen cavity of mouse was opened and exposed the orthotopic tumor in left lobe of the liver (S1B, C Fig.). The center straight one of the RITA needle was inserted into the xenograft and normal saline was dripped on the puncture site to keep good conductivity. Considering the weight and volume of nude mice, RFA was performed with a lower energy protocol, with the out power of 5W and duration of approximately 30 seconds in order to keep the existence of residual cancer. After RFA, the abdomen cavity of the mice was closed using 5-0 non-absorbable sutures. The mice in the control group were sham-operated on by inserting a RFA needle into the tumor without performing the ablation.

### Parameters Assessed

Three weeks after the RFA procedure, six mice of each group were sacrificed to evaluate tumor growth and metastasis. The longest (a) and shortest (b) tumor diameter were measured and the tumor volume was calculated as follows: Tumor volume (mm^3^)  =  a (mm) × b (mm) × b (mm)/2 [27]. The lungs were excised and images of the green fluorescent protein (GFP)-positive metastatic foci were obtained (stereomicroscope; Leica, Wetzlar, Germany). The lung tissues were sectioned serially and H&E staining was performed to confirm the above results. Intraperitoneal metastasis was observed by fluorescent imaging.

### Heat Intervention *in vitro*

HCCLM3 cells and HepG2 cells were seeded into 6-well plates at a density of 5×10^4^ cells/well. After 24 h, the plates were sealed with parafilm and submerged in a water bath set to the target temperature for 10 min. The target temperatures for HCCLM3 cells and HepG2 cells were 39°C, 42°C, 45°C and 41°C, 44°C, 47°C, respectively. Meanwhile, the control temperature was 37°C.

### Cell Proliferation, Migration and Invasion Assay

The HCC cells were seeded in 96-well plates at a density of 3×10^3^/well. After 24 h incubation, the plates of HCCLM3 cells and HepG2 cells were sealed and heated in a water bath for 10 min at 39°C, 42°C or 45°C and 41°C, 44°C or 47°C, respectively. After incubation for 24 h, 48 h and 72 h, proliferation was measured using a Cell Counting Kit (Dojin Laboratories, Kumamoto, Japan), as previously described [28].

Cell migration and invasion were assessed by transwell assays (Corning). Briefly, 8×10^4^ cells in serum-free DMEM were seeded into the upper chamber of each well of 24-well plates containing 8.0-µm pore size membranes. DMEM containing 10% fetal bovine serum (FBS) was added to the lower chamber of each well. After 48 h, cells that had reached the underside of the membrane were stained with Giemsa (Sigma), counted at ×200 magnification in five randomly selected areas per well. The cell invasion assay was carried out similarly, except that 80 µL Matrigel (0.8 mg/mL, BD Biosciences) was added to each well 6 h before cells were seeded on the membrane.

### Cell Morphological Observation and Confocal Immunofluorescence Staining

The morphologies of the HCC cells were observed using phase microscopy (Leica). The immunofluorescence staining was carried out as previously described with some changes [29]. Cultured cells were grown in cell culture dishes and then washed and fixed. Cells were then incubated with primary antibodies to E-cadherin, N-cadherin, vimentin and β-catenin (1∶200, Abcam, Hong Kong), and anti-mouse FITC, anti–rabbit FITC and/or tetramethyl rhodamine isothiocyanate–conjugated secondary antibodies (Invitrogen). The fluorescent images were taken using a confocal microscope (Olympus).

### Transient Transfection of siRNAs

To further examine the functional role of β-catenin in heat-induced malignant behavior of HCCLM3 cells, transient transfection was performed in HCCLM3 cells with small-interfering RNA (siRNA) targeting CTNNB1 mRNA (siCTTNB1, Gene Parma, Shanghai, China) or mock transfection (Gene Parma). Cells were transfected with either a control or a siRNA using Lipofectamine 2000 (Invitrogen) in OPRI-MEM medium (Gibco) according to the manufacturer's instructions. The sequence of β-catenin siRNA was: 5′GGGUUCAGAUGAUAUAAAUTT3′.

### Real-time Quantitative Reverse Transcription–polymerase Chain Reaction (RT-PCR)

The RT-PCR procedures used were described elsewhere [30]. The primer sequences used to determine the expression of the target genes are as follows: Snail, 5′-TGCAGGACTCTAATCCAAGTTTACC-3′ (forward), and Snail, 5′-GTGGGATGGCTGCCAGC-3′ (reverse); Slug, 5′-GGTCAAGAAGCATTTCAAC-3′ (forward), and Slug, 5′-CTGAGCCACTGTGGTCCTTG-3′ (reverse); Twist, 5′-TGTCCGCGTCCCACTAGC-3′ (forward), and Twist, 5′-TGTCCATTTTCTCCTTCTCTGGA-3′ (reverse).

### Western Blot Assay

The western blot procedures used are described elsewhere [31]. Primary antibodies used including: anti-E-cadherin, anti-N-cadherin, anti-vimentin, anti-β-catenin, anti-Cyclin-D1 (Abcam) and anti-β-actin (Boster, Wuhan, China).

### Immunohistochemistry

Tumor tissue was fixed, embedded and sliced into 5 µm thick sections. Immunohistochemistry staining of E-cadherin, N-cadherin, vimentin and β-catenin was carried out as described previously [30]. Staining results were evaluated under a light microscope at a magnification of ×200.

### Statistical Analysis

Statistical comparisons were performed using the Student's *t* test when data were normally distributed or the nonparametric analyses of Mann-Whitney U-test when data were not normally distributed. Calculations were made using SPSS 20.0 (SPSS Inc. Chicago, IL, USA). Results were considered statistically significant at a *p* value of <0.05.

## Results

### Heat intervention promoted proliferation, invasion and migration of HCC cell *in vitro*

**Figure 1:**
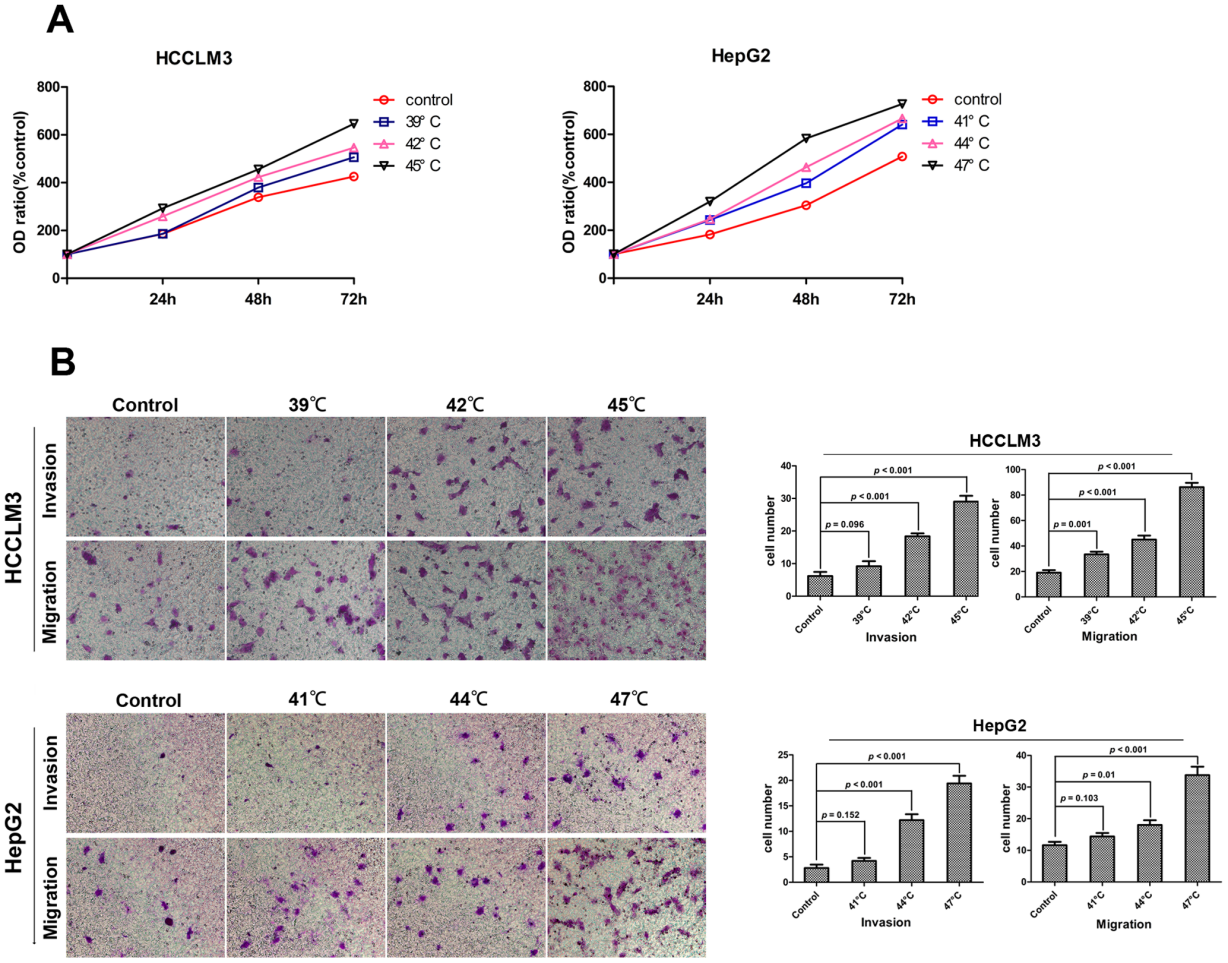
Heat intervention promoted proliferation, invasion and migration of HCC cells *in vitro*. (A) HCCLM3 and HepG2 cells were cultured with or without heat intervention. The 24 h, 48 h and 72 h cell viability of HCC cells were measured using CCK8 assay. Heat intervention significantly promoted HCCLM3 and HepG2 cells proliferation. (B) The transwell assay demonstrated that the heat-treated HCCLM3 and HepG2 cells showed enhanced migration and invasion ability compared with the controls.

An *in vitro* proliferation assay demonstrated that the heat-treated HCC cells had significantly higher proliferation than that in control (Fig. 1A), especially at 45°C for HCCLM3 cell and 47°C for HepG2 cell. In the cell migration assay, all the heat-treated HCCLM3 cells exhibited significantly higher migration potential than that in control group. (33.40±2.16, 45.00±3.21, 86.20±3.39 *vs.* 19.00±2.00), the HepG2 cells treated with 44°C and 47°C showed higher migration potential than that in control (18.00±1.52, 33.80±2.63 *vs.* 11.60±1.07) (Fig. 1B). In the cell invasion assay, the HCCLM3 cells treated with 42°C and 45°C demonstrated the enhanced invasive ability (18.40±0.87, 29.00±1.82 *vs.* 5.60±1.03), the HepG2 cells treated with 44°C and 47°C exhibited significantly higher invasive ability (12.20±1.56, 19.40±1.50 *vs.* 2.80±0.67) (Fig. 1B).

### Heat intervention confers mesenchymal characteristics to HCC cells *in vitro*

**Figure 2:**
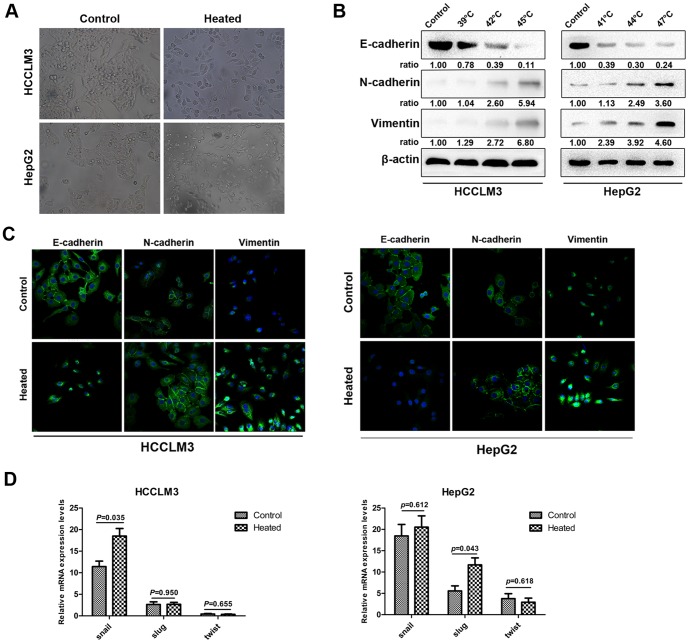
HCC cells exhibited epithelial–mesenchymal transition (EMT) after heat intervention *in vitro*. (A) HCCLM3 cells and HepG2 cells were cultured after 45°C and 47°C heat intervention, respectively. Morphologic changes consistent with EMT (spindle-shaped cells with loss of polarity and increased intercellular separation) were observed in heat-treated HCC cells in 48 h, compared with those in control. (B) Western Blot analysis revealed the expression of E-cadherin, N-cadherin, vimentin in heat-treated HCC cells and in controls. HCCLM3 cells and HepG2 cells were cultured 48 h after 45°C and 47°C heat intervention, respectively. Immunofluorescence staining (C) showed that the changes in cellular EMT markers in response to heat intervention as characterized by down-regulation of E-cadherin and up-regulation of N-cadherin and vimentin, compared with controls. RT-PCR (D) revealed that the mRNA expression of EMT related transcription factors Snail, Slug, and Twist in HCC cells.

The heat-treated HCC cells might also gain an EMT-like phenotype, morphologically, the HCCLM3 cells and HepG2 cells at 48 h after heat intervention of 45°C and 47°C, respectively, showed an irregular fibroblast-like shape instead of a typical epithelial cobblestone appearance (Fig. 2A). Meanwhile, western blot (Fig. 2B) and immunofluorescence staining (Fig. 2C) demonstrated a reduction in the expression of the epithelial cell marker E-cadherin in these heat-treated cells, compared with the control cells. At the same time, the enhanced expression of the mesenchymal cell markers N-cadherin and vimentin were also detected. Additionally, the mRNA expression of EMT transcription factors (Snail, Slug and Twist) were detected by RT-PCR. The amplification of Snail mRNA in heat-treated HCCLM3 cells and Slug mRNA in heat-treated HepG2 cells were statistically higher than those of the control (Fig. 2D).

### Incomplete RFA inhibited tumor growth but promoted invasiveness and distant metastasis *in vivo*

**Figure 3:**
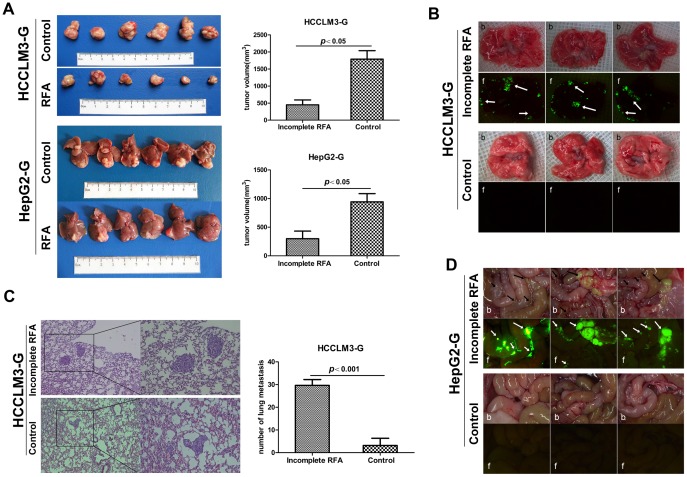
Incomplete RFA inhibited tumor growth but enhanced invasiveness and metastasis *in vivo*. (A) In the orthotopic HCC model, the tumor sizes in the incomplete RFA group were smaller than those in control (HCCLM-G: 449.58±143.19 *vs.* 1788.75±248.53, *P* = 0.002; HepG2-G: 299.83±131.45 *vs.* 942.67±144.16, *P* = 0.008). (B) Quantification of bioluminescence showed that incomplete RFA accelerated pulmonary metastasis in HCCLM3-G xenografts, compared with the matched controls (arrows indicate pulmonary metastases). Three tumors from controls and three incomplete RFA treated tumors were compared by both bright field (b) and fluorescence (f). The incidence of pulmonary metastasis (6/6) was increased in the incomplete RFA group, compared with the control group (2/6). (C) H&E staining confirmed that incomplete RFA induced more pulmonary metastases in the HCCLM3-G xenografts (29.67±2.56 *vs.* 2.50±1.58, *P*<0.001). (D) Quantification of bioluminescence showed that incomplete RFA could induce intraperitoneal metastasis in HepG2-G xenografts (6/6), compared with the matched controls (0/6) (arrows indicate intraperitoneal metastases).

We have developed a safe and reliable method to establish an incomplete RFA orthotopic nude mouse model and this animal model may be the first reported incomplete RFA orthotopic nude mouse model according to the literature in PubMed. In incomplete RFA group, the tumor size of HCCLM3-G and HepG2-G model were 449.58±143.19 mm^3^ and 299.83±131.45 mm^3^, respectively, which were smaller than those of the controls (1788.75±248.53 mm^3^ in HCCLM3-G and 942.67±144.16 mm^3^ in HepG2-G, *P*<0.05)(Fig. 3A). The pulmonary metastasis rate in the incomplete RFA group of HCCLM3-G (6/6) was higher than that in control group (2/6) (Fig. 3B). To further evaluate the metastatic potential of residual cancer of HCCLM3-G after incomplete RFA, serial lung paraffin sections were used. The pulmonary metastases in incomplete RFA group were significantly increased, compared with the control group (29.67±2.56 *vs.* 2.50±1.58, *P*<0.001) (Fig. 3C). On the other hand, pulmonary metastasis was not detected in HepG2-G model, regardless of receiving RFA or not (S2 Fig.), however, incomplete RFA could induce peritoneal metastasis (6/6), and no peritoneal metastasis was observed in control group (0/0) (Fig. 3D).

### Incomplete RFA induced changes consistent with EMT in HCC xenografts

**Figure 4:**
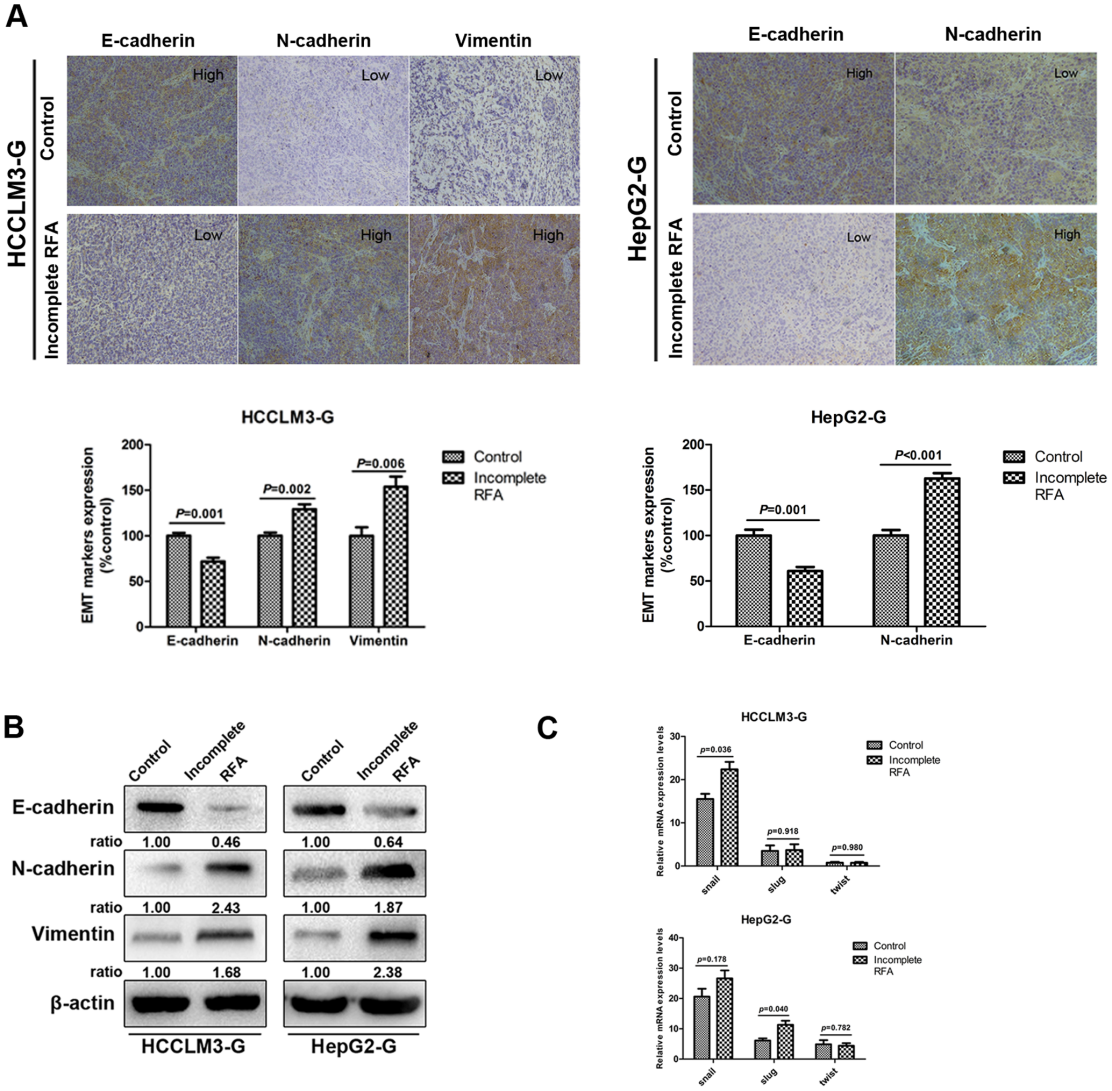
Residual tumors after incomplete RFA exhibited changes consistent with EMT. (A) Immunohistochemistry revealed that E-cadherin expression was decreased in incomplete RFA group of both HCC cell models, the expression level of N-cadherin in tumor tissues were higher in incomplete RFA group than that in controls, and in HCCLM3-G model, increased expression of vimentin was also detected in incomplete RFA group. (B) Western blot showed the changes of residual tumors were consistent with EMT in incomplete RFA group (e.g., down-regulation of E-cadherin and up-regulation of N-cadherin, vimentin) of both HCC cell models. (C) RT-PCR showed the expression of transcription factors Snail, Slug, and Twist at the mRNA level in tumor tissue. However, only Snail mRNAs in HCCLM3-G model and Slug mRNA in HepG2-G model were significantly up-regulated in incomplete RFA group, respectively (*P*<0.05).

Immunohistochemistry exhibited the typical membranous E-cadherin expression in the cell-cell contacts in control group of HCCLM3-G, in contrast, incomplete RFA-treated tumors showed the reduction of E-cadherin expression, as well as the up-regulation of N-cadherin and vimentin. In HepG2-G model, the down-regulation of E-cadherin expression and up-regulation of N-cadherin expression were also observed in incomplete RFA group (Fig. 4A). Induction of EMT by incomplete RFA were demonstrated in xenograft tumors of both cell lines by western blot, and were characterized by the loss of E-cadherin and up-regulation of N-cadherin and vimentin in the tumor tissues of incomplete RFA group (Fig. 4B). Moreover, the enhanced mRNA expression of EMT transcription factor Snail and Slug were examined by RT-PCR in HCCLM3-G-derived and HepG2-G-derived xenografts, respectively (Fig. 4C).

### Incomplete RFA induced β-catenin activating accompanied with EMT *in vivo* and *in vitro* of HCCLM3

**Figure 5:**
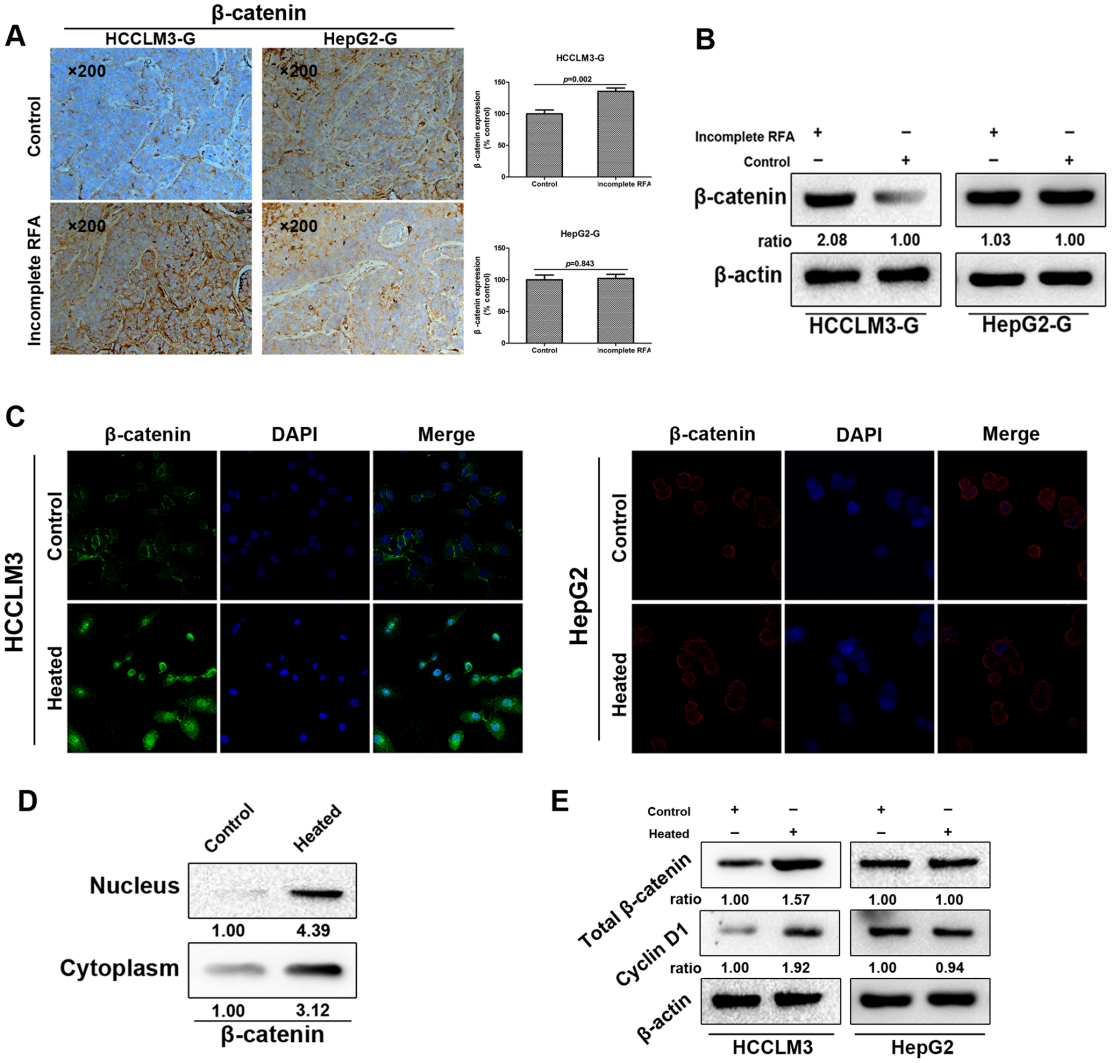
Incomplete RFA induced overexpression or internalization of β-catenin accompanied with EMT *in vivo* and *in vitro* of HCCLM3 cells. (A) Immunohistochemistry revealed that β-catenin expression was increased in incomplete RFA group of HCCLM3-G xenografts (*P* = 0.002); in HepG2-G xenografts, there was no significant difference between two groups in β-catenin expression (*P* = 0.843). (B) Western blot showed the enhanced β-catenin expression in the tumor tissues after incomplete RFA compared with the controls of HCCLM3-G xenografts. (C) Immunofluorescence staining showed the strong cytoplasmic and nuclear localization of β-catenin in heat-treated HCCLM3 cells, and the typical membranous β-catenin expression in the cell-cell contacts of HCCLM3 cells in control. However, there was no significant difference between two groups in β-catenin expression of HepG2 cells. (D) Distribution of β-catenin in cytoplasm and nucleus of HCCLM3 cells was detected by western blot *in vitro*. (E) Western blot analysis exhibited the expression of total β-catenin and its downstream target gene Cyclin-D1 of both two HCC cells *in vitro*.

To explore the molecular mechanisms involved in the EMT of heat-treated HCC cells, we attempted to test whether β-catenin signaling was activated. In the HCCLM3-G model, immunohistochemistry staining revealed a significantly enhanced intracellular accumulation of β-catenin in incomplete RFA-treated tumor, but not in HepG2-G model (Fig. 5A). Those results were consistent with the β-catenin protein expression in the incomplete RFA group and the control group by western blot (Fig. 5B). Accumulation of cytoplasmic and nuclear β-catenin is an indication of activated β-catenin-dependent signaling, so we further examine whether increased β-catenin expression affected β-catenin pathway activity. Immunofluorescence staining showed the increased expression of β-catenin in both cytoplasm and nucleus of the heat-treated HCCLM3 cells, compared with the control cells *in vitro*, however, the same trend in the heat-treated HepG2 cells was not observed (Fig. 5C). In addition, a marked intracellular translocation of β-catenin in the heat-treated HCCLM3 cells was verified by western blot analysis (Fig. 5D). To examine the effect of heat intervention inducing nuclear translocation of β-catenin on gene expression, we turned attention to the β-catenin signaling pathway downstream target gene Cyclin-D1. Western blot revealed that the expression of Cyclin-D1 was also increased with total β-catenin overexpression in the heat-treated HCCLM3 cells *in vitro* (Fig. 5E).

### The enhanced invasion potential and EMT-like changes of heat-treated HCCLM3 cells can be partially attenuated by silencing β-catenin *in vitro*

**Figure 6:**
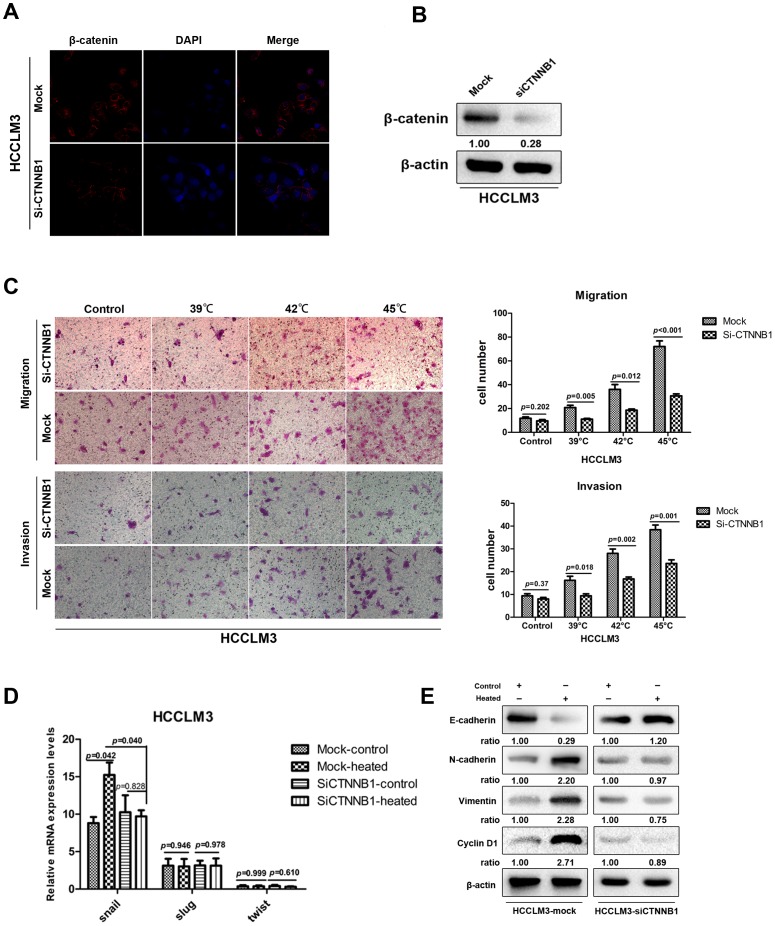
The effects induced by heat intervention were partially attenuated by silencing β-catenin in HCCLM3 cells. (A) Immunofluorescence staining indicated that β-catenin expression was partially attenuated by silencing of CTNNB1 in HCCLM3 cells. (B) The protein levels of β-catenin measured by western blot analysis after siCTNNB1 transfection. (C) Representative images of migration and invasion of HCCLM3-mock cells and HCCLM3-siCTNNB1 cells in transwell assay. HCCLM3-mock cells and HCCLM3-siCTNNB1 cells were cultured 48 h after 45°C heat intervention and normal condition, respectively, RT-PCR (D) showed the relative mRNA expression of transcription factors Snail, Slug, and Twist. Western blot (E) revealed the expression of EMT related transcription factors and Cyclin-D1.

To further elucidate whether the activation of β-catenin was functionally associated with enhanced invasiveness and EMT-like changes in heat-treated HCCLM3 cells, we used CTNNB1 siRNA transfection and examined its effects on HCCLM3 cells. In order to check the effects of transfection, immunofluorescence staining and western blot were used. As shown in Fig. 6A, the typical cell membrane expression of β-catenin was reduced after transfection, and the total β-catenin protein was decreased in HCCLM3-siCTNNB1 cells, compared with the control of HCCLM3-mock cells (Fig. 6B). As shown in Fig. 6C, under normal conditions, the difference in penetrating cell numbers between HCCLM3-mock cells and HCCLM3-siCTNNB1 cells were not statistically significant. However, after the introduction of heat intervention, the number of HCCLM3-siCTNNB1 cells in both migration array and invasion array were less than those of HCCLM3-mock cells (Fig. 6C). Furthermore, the silencing of CTNNB1 could influence the expression of EMT transcription factor, RT-PCR revealed that the expression of Snail mRNA of heat-treated HCCLM3-siCTNNB1 cells was decreased, compared with the counterpart in heat-treated HCCLM3-mock cells (Fig. 6D). Western blot analysis also confirmed that repression of β-catenin affected the expression of β-catenin-mediated EMT markers in heat-treated HCCLM3 cells, and the expression of Cyclin-D1 was also attenuated (Fig. 6E). Collectively, the above findings demonstrated that activation of β-catenin was functionally relevant to invasion and metastasis of HCCLM3 cells mediated by heat intervention.

## Discussion

At present, a growing number of clinical studies have identified the rapid progression of residual cancer after incomplete RFA in treating HCC [32], [33]. Previous researches had provided several potential mechanisms that might help explain these findings. Hyperthermia may play an important role in the rapid growth of residual HCC after RFA by promoting angiogenesis of residual HCC via HIF-1a/VEGFA [34]. Another study demonstrated that suboptimal RFA accelerated HCC growth and spread by transiently inducing an EMT-like and more aggressive cellular phenotype [35]. In the present study, we introduced RFA into an orthotopic nude mouse HCC model and demonstrated the significance of incomplete RFA treatment on tumor growth and invasiveness. Although the xenograft tumor volumes were reduced after incomplete RFA treatment, the residual tumors showed more invasive abilities in incomplete RFA-treated mice, as indicated by increased pulmonary or intraperitoneal metastasis. On the other hand, *in vitro* heat-treated HCC cells exhibited increased motility and invasiveness. These findings were consistent with the results of previous studies using other animal cancer models. In a translational murine model, Kroeze et al. identified that incomplete thermal ablation stimulated proliferation of residual renal carcinoma cells [36]. Ke et al. found residual hepatic VX2 carcinoma after incomplete RFA could facilitate its rapid progression in a VX2 carcinoma rabbit model, and the focal tumor volume and lung metastases of RFA-treated rabbits significantly increased [37].

Mounting evidence had identified a significant correlation between EMT and invasiveness in various types of tumor [38]–[40]. Herein we showed that heat intervention stimulated transformation of HCC cells from a typical epithelial phenotype to a spindle-shaped mesenchymal phenotype, accompanied by the down-regulation of E-cadherin and up-regulation of N-cadherin and vimentin. Further evidence provided by xenografts in nude mice after incomplete RFA was that protein level alterations of these EMT markers were also detected. Furthermore, we also examined the up-regulated EMT transcription factors in incomplete RFA tumors and in heat-treated HCC cells. These findings combined with the results of previous studies mentioned above, further identified that the “cadherin switch” of tumor tissue might be triggered by incomplete RFA which lead to enhanced metastasis potential.

To fully understand the molecular mechanism of the relation between heat intervention and the changes of cell phenotype, we focused our attention on β-catenin. As a pivotal factor in the Wnt signal pathway, β-catenin plays an important role in HCC progression, development, phenotype changes and carcinogenesis. β-catenin mutation is one factor that may be critical to the development of HCC. Mutations involving the Wnt/β-catenin pathway appear to be one of the most frequent genetic events that contributes to liver carcinogenesis and progression [41]. Dysregulation of β-catenin may promote carcinogenesis; mice heterozygous for Lkb1 deletion showed an accelerated progression to HCC when mated with adenovirus-inducible β-catenin mutant mice [42]. Meanwhile, some reports had implicated that β-catenin mutation was a rather common mechanism of mouse hepatocellular neoplasms, including adenomas and carcinomas [43]. On the other hand, the correlation between β-catenin signaling pathway and EMT has been verified in several studies. Yang et al. suggested SOX2 which was a high mobility group box containing transcription factor essential for the maintenance of embryonic stem cells could modulate invasion and EMT of human laryngeal cancer cell line Hep-2 through Wnt/β-catenin signaling pathway [44]. Another study also proved that stromal cell-derived factor 1 (SDF-1) and its receptor, CXCR4, promoted colorectal cancer progression and EMT by activation of β-catenin [45].

Based on the above studies, we hypothesized that heat intervention might activate β-catenin, and then the activated β-catenin contributed to the cadherin switching in the heat-treated HCC cells. In the present study, the effects of heat intervention on β-catenin were examined and elevated protein levels and/or intra-nuclear accumulation of β-catenin were verified in HCCLM3 cells *in vitro*. The key downstream gene Cyclin-D1 in Wnt/β-catenin signaling was also up-regulated. Using *in vivo* incomplete RFA HCC models, we further demonstrated that the aberrant activation of β-catenin was positively correlated with RFA-induced invasion and metastasis in HCCLM3-G model. However, the similar trend in HepG2 cell line was not observed, regardless of *in vivo* or *in vitro*, there might be other molecular mechanisms that promoted cadherin switching and some of the pathways downstream of this process that influenced HepG2 cell aggressive behaviors. Future researches will be also needed to further understanding the underlying mechanism of cadherin switching of HepG2 cells.

This study clearly demonstrated that heat intervention might directly enhance the invasiveness of HCCLM3 cells by EMT via activating β-catenin and increasing Snail mRNA expression. The following evidences support this conclusion. First, incomplete RFA not only significantly heightened the level of β-catenin in the nucleus, but also promoted the EMT transcription factor Snail mRNA expression. Moreover, a positive relationship between EMT and nuclear β-catenin accumulation was found in heat-treated HCCLM3 cells *in vitro*, and EMT was also found in incomplete RFA tumor tissues accompanying the up-regulation of β-catenin protein expression. Second, silencing of β-catenin not only significantly decreased expression of downstream target gene Cyclin-D1 and transcription factor Snail, but also attenuated EMT of heat-treated HCCLM3 cells.

In our study, we used the incomplete RFA orthotopic HCC model to identify the invasiveness and metastasis of residual cancer. According to the relevant literature, the previous researches on biological behavior of residual cancer after RFA treatment were based on nude mice subcutaneous xenograft models or rabbit orthotopic models, there was no report about research using orthotopic HCC nude mouse model [37], [46]–[48]. Actually, as a mature HCC animal model, orthotopic nude mice models can do a better job in RFA research with imitating the *in vivo* environment, especially in studying the invasive and metastatic abilities of HCC [49], [50].

In conclusion, the findings of this study using an orthotopic HCC model shed light on the enhanced invasive abilities of residual cancer after incomplete RFA and demonstrated the significant role of β-catenin and EMT. These data from animal experiments also need further investigation of RFA treatment in humans.

## Supporting Information

S1 Fig **Equipment that used in establishing incomplete RFA orthotopic nude mouse model.** (A) The retractable multiple hook RFA needle was located in the top half of the picture, the middle straight needle electrode remarked by the red circle was used during the RFA process. (B) Opening the abdominal cavity and the xenograft tumor in the left liver lobe was fully exposed. Black arrow: the xenograft tumor. (C) The retractable RFA needle was extended and the middle straight thin needle was inserted into the xenograft tumor during RFA process.
(TIF)

S2 Fig **Quantification of bioluminescence evaluated the pulmonary metastasis in HepG2-G othotopic model.** Pulmonary metastasis was not detected in both incomplete RFA group and control group of HepG2-G model.
(TIF)

S1 Checklist **The ARRIVE (Animal Research: Reporting In Vivo Experiments) Guidelines Checklist.**
(PDF)
