# Correction: Incomplete Radiofrequency Ablation Enhances Invasiveness and Metastasis of Residual Cancer of Hepatocellular Carcinoma Cell HCCLM3 via Activating β-Catenin Signaling

**DOI:** 10.1371/journal.pone.0120472

**Published:** 2015-03-16

**Authors:** The PLOS ONE Staff

The following information is missing from the Funding section: This study was supported by the National S&T Major Project for Infectious Diseases of China (NO.2012ZX10002–017)).

The complete, correct Funding section is as follows: This work was supported by National Natural Science Foundation of China (grant number: 81372314); The National Key Project for Infectious Diseases (2012ZX10002012–004); National S&T Major Project for Infectious Diseases of China (NO.2012ZX10002–017); Shanghai Natural Science Fund for Youth Scholars (12ZR1442300). The funders had no role in study design, data collection and analysis, decision to publish, or preparation of the manuscript.
