# Supplementary figures and images for: Incomplete Radiofrequency Ablation Enhances Invasiveness and Metastasis of Residual Cancer of Hepatocellular Carcinoma Cell HCCLM3 via Activating β-Catenin Signaling
Source: PLoS One. 2014 Dec 26;9(12):e115949. doi: 10.1371/journal.pone.0115949 (PMC4277411; doi:10.1371/journal.pone.0115949)

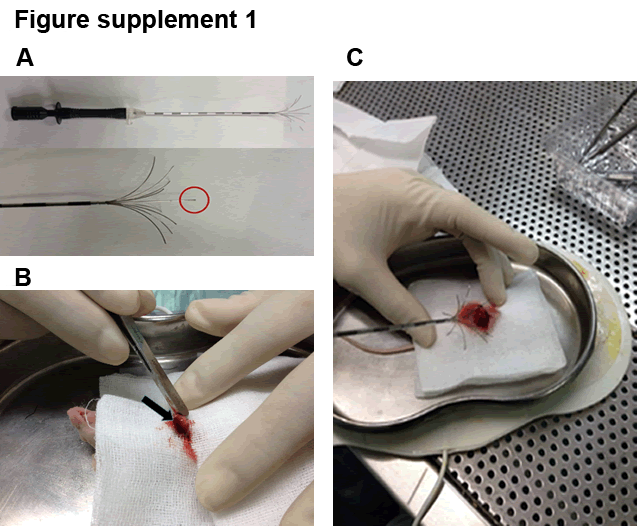

Supplement: S1 Fig — Equipment that used in establishing incomplete RFA orthotopic nude mouse model. (A) The retractable multiple hook RFA needle was located in the top half of the picture, the middle straight needle electrode remarked by the red circle was used during the RFA process. (B) Opening the abdominal cavity and the xenograft tumor in the left liver lobe was fully exposed. Black arrow: the xenograft tumor. (C) The retractable RFA needle was extended and the middle straight thin needle was inserted into the xenograft tumor during RFA process. (TIF) [file pone.0115949.s001.tif]

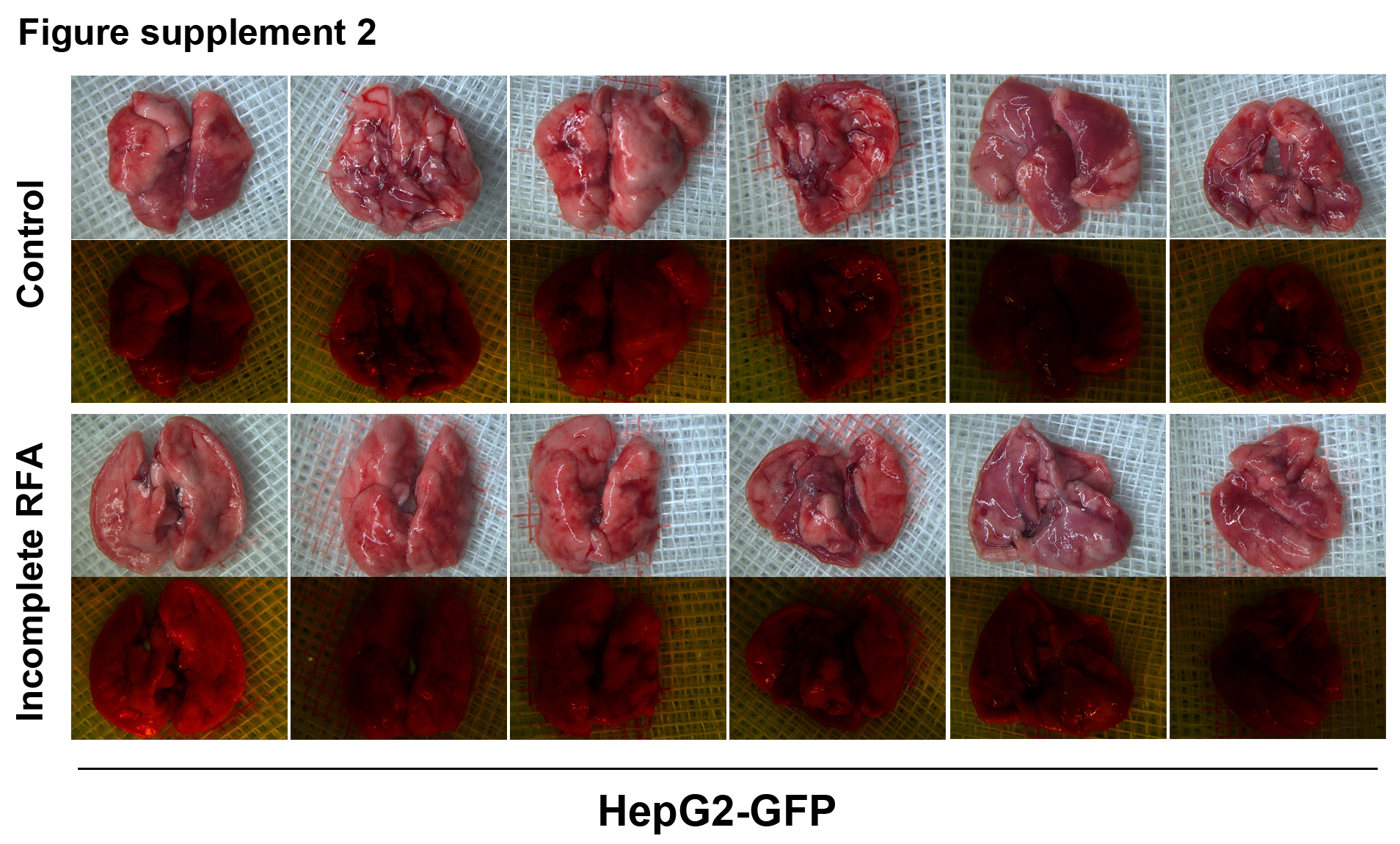

Supplement: S2 Fig — Quantification of bioluminescence evaluated the pulmonary metastasis in HepG2-G othotopic model. Pulmonary metastasis was not detected in both incomplete RFA group and control group of HepG2-G model. (TIF) [file pone.0115949.s002.tif]
